# Crystal structure of *N*-de­acetyl­lappa­coni­tine

**DOI:** 10.1107/S2056989015012335

**Published:** 2015-07-15

**Authors:** Xin-Wei Shi, Qiang-Qiang Lu, Jun-Hui Zhou, Xin-Ai Cui

**Affiliations:** a Xi’an Botanical Garden, Institute of Botany of Shaanxi Province, Xi’an 710061, People’s Republic of China

**Keywords:** *N*-de­acetyl­lappaconitine, C_19_-diterpenoid alkaloid, O—H⋯O hydrogen bonding., crystal structure

## Abstract

The title compound, C_30_H_42_N_2_O_7_ [systematic name: (1*S*,4*S*,5*S*,7*S*,8*S*,9*S*,10*S*,11*S*,13*R*,14*S*,16*S*,17*R*)-20-ethyl-4,8,9-trihy­droxy-1,14,16-tri­meth­oxy­aconitan-4-yl 2-amino­benzoate], isolated from roots of *Aconitum sinomontanum* Nakai, is a typical aconitane-type C_19_-diterpenoid alkaloid, which crystallizes with two independent mol­ecules in the asymmetric unit. The conformations of the two independent mol­ecules are closely similar. Each mol­ecule comprises four six-membered rings (*A*, *B*, *D* and *E*) including one six-membered N-containing heterocyclic ring (*E*), and two five-membered rings (*C* and *F*). Rings *A*, *B* and *E* adopt chair conformations, while ring *D* displays a boat conformation. Five-membered rings *C* and *F* exhibit envelope conformations. IntramolecularN—H⋯O hydrogen bonds between the amino group and carbonyl O atom help to stabilize molecular structure. In the crystal, O—H⋯O hydrogen bonds link the mol­ecules into zigzag chains propagating in [010].

## Related literature   

For reviews of typical C_19_-diterpenoid alkaloids, see: Wang *et al.* (2009 ▸, 2010 ▸). For the isolation, idenfication and biological activity of *N*-de­acetyl­lappaconitine, see: Peng *et al.* (2000 ▸); Romanov *et al.* (2008 ▸). For ring numbering, ring conformations and absolute configurations of C_19_-diterpenoid alkaloids, see: Wang *et al.* (2007 ▸); He *et al.* (2008 ▸).
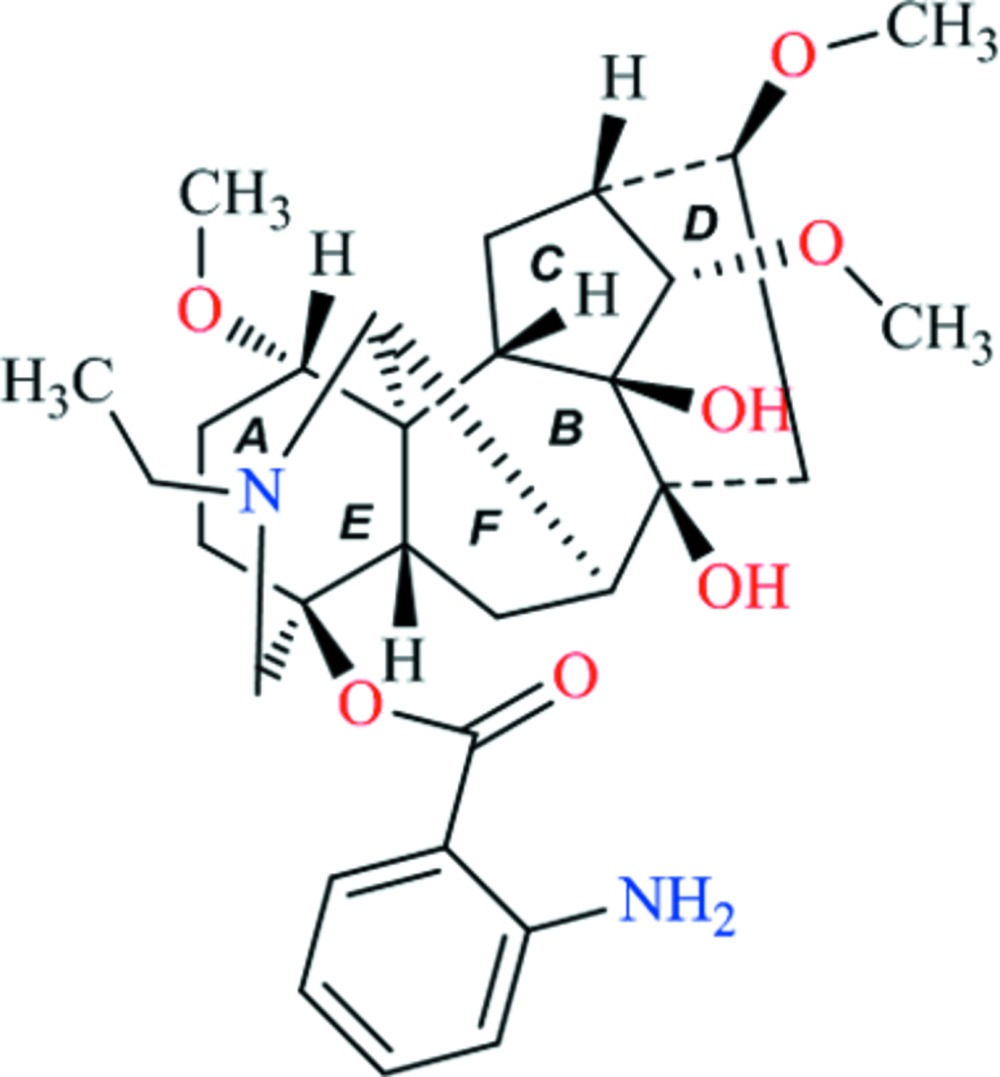


## Experimental   

### Crystal data  

C_30_H_42_N_2_O_7_
*M* *_r_* = 542.66
Orthorhombic, 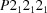

*a* = 11.7090 (3) Å
*b* = 13.2040 (4) Å
*c* = 35.7380 (9) Å
*V* = 5525.3 (3) Å^3^
*Z* = 8
Cu *K*α radiation
μ = 0.75 mm^−1^
*T* = 173 K
0.30 × 0.30 × 0.30 mm

### Data collection  

Bruker SMART CCD area-detector diffractometer
Absorption correction: multi-scan (*SADABS*; Bruker, 2002 ▸) *T*_min_ = 0.806, *T*_max_ = 0.806
18595 measured reflections
8477 independent reflections
7744 reflections with *I* > 2σ(*I*)
*R*_int_ = 0.030

### Refinement  

*R*[*F* ^2^ > 2σ(*F* ^2^)] = 0.042
*wR*(*F* ^2^) = 0.111
*S* = 1.02
8477 reflections
713 parameters
H-atom parameters constrained
Δρ_max_ = 0.26 e Å^−3^
Δρ_min_ = −0.19 e Å^−3^

### 

Data collection: *SMART* (Bruker, 2002 ▸); cell refinement: *SAINT*; data reduction: *SAINT* (Bruker, 2002 ▸); program(s) used to solve structure: *SHELXTL* (Sheldrick, 2008 ▸); program(s) used to refine structure: *SHELXTL*; molecular graphics: *SHELXTL* and *Mercury* (Macrae *et al.*, 2008 ▸); software used to prepare material for publication: *SHELXTL*.

## Supplementary Material

Crystal structure: contains datablock(s) I, New_Global_Publ_Block. DOI: 10.1107/S2056989015012335/cv5490sup1.cif

Structure factors: contains datablock(s) I. DOI: 10.1107/S2056989015012335/cv5490Isup2.hkl

Supporting information file. DOI: 10.1107/S2056989015012335/cv5490Isup4.cdx

**Fig. 1.**
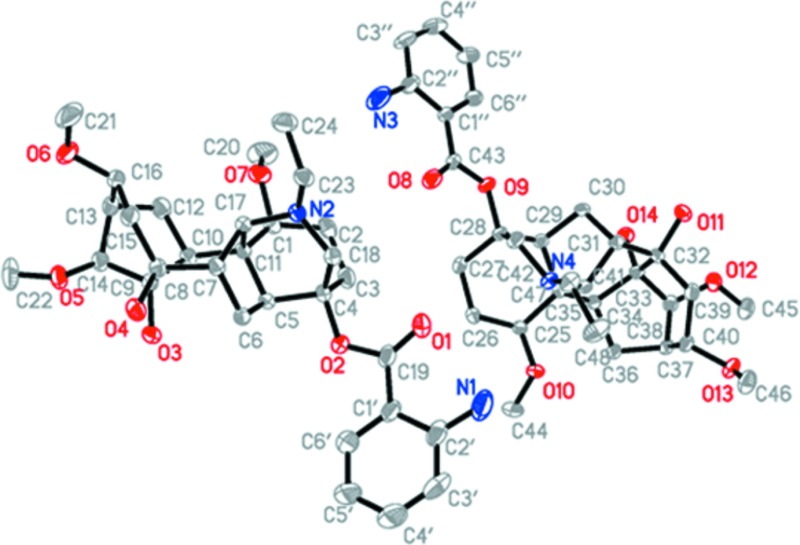
Two independent molecules in the asymmetric unit showing the atomic labeling and 30% probabilty displacement ellipsoids. H atoms omitted for clarity.

. DOI: 10.1107/S2056989015012335/cv5490fig1.tif
Two independent mol­ecules in the asymmetric unit showing the atomic labeling and 30% probabilty displacement ellipsoids. H atoms omitted for clarity.

**Fig. 2.**
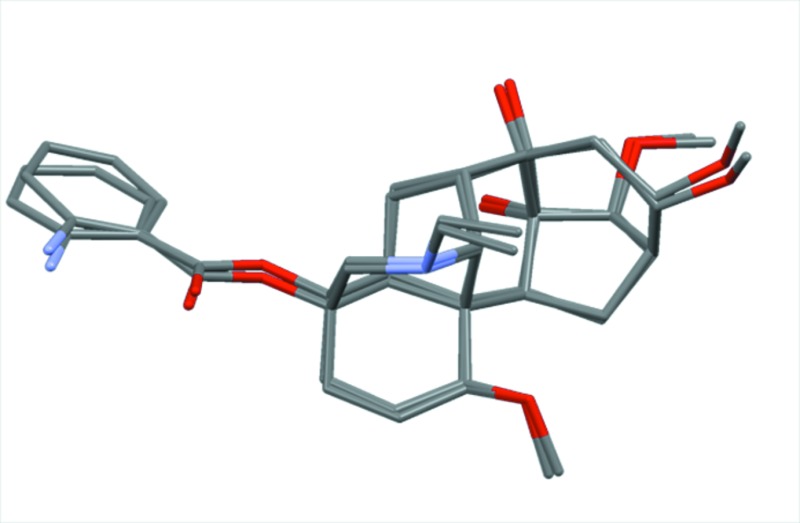
The overlay of two independent molecules.

. DOI: 10.1107/S2056989015012335/cv5490fig2.tif
The overlay of two independent mol­ecules.

CCDC reference: 1409115

Additional supporting information:  crystallographic information; 3D view; checkCIF report

## Figures and Tables

**Table 1:** Hydrogen-bond geometry (, )

*D*H*A*	*D*H	H*A*	*D* *A*	*D*H*A*
O14H14*A*O6^i^	0.98	2.27	2.927(2)	123
O11H11O12	0.84	2.40	2.944(2)	124
O4H4O5	0.84	2.33	2.914(3)	127
O3H3O13^ii^	0.84	2.41	3.095(2)	139
N3H3*D*O8	0.91	2.02	2.687(3)	129
N1H1*A*O1	0.91	2.02	2.752(4)	137

Symmetry codes: (i) 
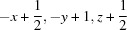
; (ii) 
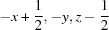
.

## supplementary crystallographic information

### S1. Comment

The title compound, *N*-de­acetyl­lappaconitine, is produced by several species of the plant genus *Aconitum* (*A. sinomontanum*, *A. barbatum*, *A. septentrionale*, *A. leucostomum*, *A. orientale*) and its structure was confirmed by the NMR and MS data. It possesses anti­arrhythmic, analgesic, local anesthetic, sedative and anti-inflammatory activity (Peng *et al.* 2000; Wang *et al.* 2009, 2010; Romanov *et al.* 2008). Herewith we present the crystal structure of *N*-de­acetyl­lappaconitine (I).

The title compound (I) crystallizes with two independent molecules in the asymmetric unit (Fig. 1). The confomations of both molecules are close (Fig. 2) due to intra­molecular N—H···O and O—H···O hydrogen bonds (Table 1). Each molecule is composed from six rings labelled as *A*-*F* (Wang *et al.*, 2007). Six-membered rings *A* (C1–C5/C11) and *B* (C7–C11/C17) adopt chair conformations; six-membered N-containing heterocyclic ring *E* (C4/C5/C11/C17/N2/C18) display the same chair conformation; the five-membered rings *C* (C9/C10/C12/C13/C14) and *F* (C5/C6/C7/C17/C11) form envelope conformations, in which, atoms C13 and C17, respectively, play the role of flap. The six-membered ring *D* (C8/C9/C14/C13/C16/C15) is in a boat conformation.

In the crystal, inter­molecular O—H···O hydrogen bonds (Table 1) link the molecules into zigzag chains propagated in [010] .

### S2. Experimental

The title compound was isolated from the roots of *Aconitum sinomontanum* Nakai following the known procedure (Peng *et al.*, 2000). Colourless single crystals suitable for X-ray diffraction were obtained by slow evaporation from a methanol solution for two weeks at the room temperacture.

### S3. Refinement

The hydrogen atoms were placed in calculated positions and refined as riding with U_iso_(H) =1.2–1.5 U_eq_ (C, O). The positions of methyl and hy­droxy hydrogens were rotationally optimized. In spite of acceptable value of Flack parameter of -0.07 (15) in the abscence of anomalous scatterers, the absolute configuration of the title compound has been assigned to be the same as that reported for typical natural aconitane-type C_19_-diterpenoid alkaloids (Wang *et al.*, 2007; He *et al.*, 2008).

### Crystal data

**Table:**

C_30_H_42_N_2_O_7_	*F*(000) = 2336
*M**_r_* = 542.66	*D*_x_ = 1.305 Mg m^−^^3^
Orthorhombic, *P*2_1_2_1_2_1_	Cu *K*α radiation, λ = 1.54184 Å
Hall symbol: P 2ac 2ab	Cell parameters from 7732 reflections
*a* = 11.7090 (3) Å	θ = 3.6–66.9°
*b* = 13.2040 (4) Å	µ = 0.75 mm^−^^1^
*c* = 35.7380 (9) Å	*T* = 173 K
*V* = 5525.3 (3) Å^3^	Prism, colourless
*Z* = 8	0.30 × 0.30 × 0.30 mm

### Data collection

**Table:**

Bruker SMART CCD area-detector diffractometer	8477 independent reflections
Radiation source: fine-focus sealed tube	7744 reflections with *I* > 2σ(*I*)
Graphite monochromator	*R*_int_ = 0.030
phi and ω scans	θ_max_ = 65.0°, θ_min_ = 2.5°
Absorption correction: multi-scan (*SADABS*; Bruker, 2002)	*h* = −13→13
*T*_min_ = 0.806, *T*_max_ = 0.806	*k* = −12→15
18595 measured reflections	*l* = −42→38

### Refinement

**Table:**

Refinement on *F*^2^	Primary atom site location: structure-invariant direct methods
Least-squares matrix: full	Secondary atom site location: difference Fourier map
*R*[*F*^2^ > 2σ(*F*^2^)] = 0.042	Hydrogen site location: inferred from neighbouring sites
*wR*(*F*^2^) = 0.111	H-atom parameters constrained
*S* = 1.02	*w* = 1/[σ^2^(*F*_o_^2^) + (0.0677*P*)^2^ + 0.5213*P*] where *P* = (*F*_o_^2^ + 2*F*_c_^2^)/3
8477 reflections	(Δ/σ)_max_ = 0.001
713 parameters	Δρ_max_ = 0.26 e Å^−^^3^
0 restraints	Δρ_min_ = −0.19 e Å^−^^3^

### Special details

**Table:**

Geometry. All e.s.d.'s (except the e.s.d. in the dihedral angle between two l.s. planes) are estimated using the full covariance matrix. The cell e.s.d.'s are taken into account individually in the estimation of e.s.d.'s in distances, angles and torsion angles; correlations between e.s.d.'s in cell parameters are only used when they are defined by crystal symmetry. An approximate (isotropic) treatment of cell e.s.d.'s is used for estimating e.s.d.'s involving l.s. planes.
Refinement. Refinement of *F*^2^ against ALL reflections. The weighted *R*-factor *wR* and goodness of fit *S* are based on *F*^2^, conventional *R*-factors *R* are based on *F*, with *F* set to zero for negative *F*^2^. The threshold expression of *F*^2^ > σ(*F*^2^) is used only for calculating *R*-factors(gt) *etc*. and is not relevant to the choice of reflections for refinement. *R*-factors based on *F*^2^ are statistically about twice as large as those based on *F*, and *R*- factors based on ALL data will be even larger.

### Fractional atomic coordinates and isotropic or equivalent isotropic displacement parameters (Å^2^)

**Table:**

	*x*	*y*	*z*	*U*_iso_*/*U*_eq_	
C1	0.2568 (2)	0.43649 (19)	0.54143 (6)	0.0422 (6)	
H1	0.3404	0.4330	0.5358	0.051*	
C2	0.2385 (2)	0.3917 (2)	0.58046 (6)	0.0505 (6)	
H2A	0.3022	0.4132	0.5968	0.061*	
H2B	0.1671	0.4198	0.5911	0.061*	
C3	0.2312 (2)	0.2773 (2)	0.58093 (7)	0.0527 (7)	
H3A	0.3069	0.2479	0.5751	0.063*	
H3B	0.2081	0.2538	0.6061	0.063*	
C4	0.1451 (2)	0.24306 (19)	0.55241 (6)	0.0401 (5)	
C5	0.1853 (2)	0.26898 (18)	0.51258 (6)	0.0359 (5)	
H5	0.2602	0.2362	0.5067	0.043*	
C6	0.0911 (2)	0.23517 (18)	0.48488 (6)	0.0388 (5)	
H6A	0.1250	0.2038	0.4623	0.047*	
H6B	0.0387	0.1860	0.4968	0.047*	
C7	0.02808 (19)	0.33336 (18)	0.47485 (6)	0.0364 (5)	
H7	−0.0564	0.3221	0.4754	0.044*	
C8	0.06474 (19)	0.37144 (19)	0.43617 (6)	0.0380 (5)	
C9	0.19841 (18)	0.37717 (18)	0.43412 (6)	0.0362 (5)	
C10	0.25048 (19)	0.41825 (18)	0.47103 (6)	0.0369 (5)	
H10	0.3308	0.3927	0.4721	0.044*	
C11	0.19213 (18)	0.38589 (18)	0.50856 (6)	0.0342 (5)	
C12	0.2580 (3)	0.5349 (2)	0.46378 (7)	0.0541 (7)	
H12A	0.2160	0.5725	0.4834	0.065*	
H12B	0.3387	0.5574	0.4640	0.065*	
C13	0.2039 (2)	0.5545 (2)	0.42508 (7)	0.0503 (6)	
H13	0.2413	0.6133	0.4124	0.060*	
C14	0.2339 (2)	0.4561 (2)	0.40577 (6)	0.0454 (6)	
H14	0.3185	0.4527	0.4022	0.054*	
C15	0.0062 (2)	0.4726 (2)	0.42595 (7)	0.0480 (6)	
H15A	−0.0228	0.4664	0.4000	0.058*	
H15B	−0.0612	0.4803	0.4424	0.058*	
C16	0.0749 (3)	0.5707 (2)	0.42826 (7)	0.0531 (7)	
H16	0.0586	0.6035	0.4529	0.064*	
C17	0.06348 (18)	0.40758 (17)	0.50614 (6)	0.0336 (5)	
H17	0.0497	0.4792	0.4982	0.040*	
C18	0.0243 (2)	0.28618 (19)	0.55827 (7)	0.0415 (5)	
H18A	−0.0319	0.2410	0.5460	0.050*	
H18B	0.0070	0.2875	0.5854	0.050*	
C19	0.0950 (2)	0.0736 (2)	0.57788 (7)	0.0531 (7)	
C20	0.3107 (4)	0.6053 (3)	0.55720 (12)	0.0920 (12)	
H20A	0.3241	0.5891	0.5836	0.138*	
H20B	0.2859	0.6760	0.5550	0.138*	
H20C	0.3814	0.5956	0.5430	0.138*	
C21	−0.0699 (4)	0.6774 (3)	0.40392 (12)	0.1110 (17)	
H21A	−0.1243	0.6210	0.4037	0.167*	
H21B	−0.0878	0.7241	0.3834	0.167*	
H21C	−0.0751	0.7133	0.4278	0.167*	
C22	0.2309 (3)	0.4802 (3)	0.34001 (8)	0.0855 (11)	
H22A	0.2215	0.5538	0.3417	0.128*	
H22B	0.1949	0.4556	0.3170	0.128*	
H22C	0.3124	0.4635	0.3396	0.128*	
C23	−0.1048 (2)	0.4258 (2)	0.54681 (7)	0.0426 (5)	
H23A	−0.1374	0.4002	0.5706	0.051*	
H23B	−0.1514	0.3981	0.5261	0.051*	
C24	−0.1135 (3)	0.5397 (2)	0.54635 (9)	0.0624 (8)	
H24A	−0.0592	0.5682	0.5644	0.094*	
H24B	−0.1912	0.5600	0.5532	0.094*	
H24C	−0.0957	0.5648	0.5212	0.094*	
C25	0.39217 (18)	0.08850 (16)	0.71788 (6)	0.0320 (5)	
H25	0.4767	0.0942	0.7212	0.038*	
C26	0.3616 (2)	0.14105 (18)	0.68090 (6)	0.0408 (5)	
H26A	0.4200	0.1237	0.6619	0.049*	
H26B	0.2873	0.1148	0.6720	0.049*	
C27	0.3544 (2)	0.25473 (18)	0.68429 (6)	0.0399 (5)	
H27A	0.3261	0.2841	0.6605	0.048*	
H27B	0.4311	0.2831	0.6894	0.048*	
C28	0.27342 (18)	0.28149 (16)	0.71598 (6)	0.0313 (5)	
C29	0.32419 (18)	0.24848 (16)	0.75345 (6)	0.0309 (4)	
H29	0.3994	0.2820	0.7582	0.037*	
C30	0.2373 (2)	0.27287 (16)	0.78494 (6)	0.0364 (5)	
H30A	0.2768	0.3006	0.8072	0.044*	
H30B	0.1802	0.3228	0.7762	0.044*	
C31	0.18008 (18)	0.17164 (16)	0.79424 (6)	0.0334 (5)	
H31	0.0956	0.1804	0.7967	0.040*	
C32	0.22999 (19)	0.12554 (17)	0.83008 (6)	0.0350 (5)	
C33	0.36444 (19)	0.12486 (17)	0.82810 (6)	0.0338 (5)	
C34	0.40731 (17)	0.09500 (16)	0.78846 (6)	0.0312 (4)	
H34	0.4846	0.1263	0.7856	0.037*	
C35	0.33562 (16)	0.13118 (15)	0.75396 (5)	0.0272 (4)	
C36	0.42728 (19)	−0.02177 (18)	0.79183 (6)	0.0383 (5)	
H36A	0.3865	−0.0580	0.7716	0.046*	
H36B	0.5097	−0.0376	0.7900	0.046*	
C37	0.38056 (19)	−0.05397 (17)	0.83042 (6)	0.0361 (5)	
H37	0.4222	−0.1147	0.8401	0.043*	
C38	0.41089 (19)	0.03980 (18)	0.85266 (6)	0.0369 (5)	
H38	0.4959	0.0458	0.8539	0.044*	
C39	0.17956 (19)	0.01971 (18)	0.83848 (6)	0.0391 (5)	
H39A	0.1624	0.0166	0.8656	0.047*	
H39B	0.1059	0.0142	0.8250	0.047*	
C40	0.2514 (2)	−0.07378 (17)	0.82849 (6)	0.0366 (5)	
H40	0.2319	−0.0947	0.8024	0.044*	
C41	0.20902 (16)	0.10515 (16)	0.75957 (6)	0.0284 (4)	
H41	0.1999	0.0317	0.7657	0.034*	
C42	0.15309 (19)	0.23520 (17)	0.71190 (6)	0.0367 (5)	
H42A	0.0981	0.2770	0.7262	0.044*	
H42B	0.1304	0.2374	0.6852	0.044*	
C43	0.21456 (19)	0.45045 (17)	0.69410 (6)	0.0368 (5)	
C44	0.4515 (3)	−0.0713 (2)	0.69438 (9)	0.0706 (9)	
H44A	0.4605	−0.0428	0.6692	0.106*	
H44B	0.4286	−0.1425	0.6924	0.106*	
H44C	0.5242	−0.0667	0.7079	0.106*	
C45	0.4057 (3)	−0.0248 (2)	0.91444 (7)	0.0575 (7)	
H45A	0.3667	−0.0890	0.9092	0.086*	
H45B	0.3901	−0.0041	0.9403	0.086*	
H45C	0.4882	−0.0337	0.9110	0.086*	
C46	0.1203 (2)	−0.2031 (2)	0.84451 (8)	0.0578 (7)	
H46A	0.0578	−0.1553	0.8490	0.087*	
H46B	0.1102	−0.2630	0.8604	0.087*	
H46C	0.1200	−0.2236	0.8182	0.087*	
C47	0.02761 (19)	0.09681 (19)	0.72501 (7)	0.0428 (6)	
H47A	−0.0084	0.1189	0.7013	0.051*	
H47B	−0.0132	0.1307	0.7458	0.051*	
C48	0.0124 (2)	−0.0161 (2)	0.72891 (10)	0.0645 (8)	
H48A	0.0617	−0.0509	0.7109	0.097*	
H48B	−0.0675	−0.0339	0.7241	0.097*	
H48C	0.0332	−0.0368	0.7543	0.097*	
C1'	0.0897 (2)	−0.0336 (2)	0.56605 (8)	0.0553 (7)	
C2'	0.0381 (2)	−0.1087 (3)	0.58835 (9)	0.0642 (8)	
C3'	0.0275 (3)	−0.2063 (3)	0.57384 (13)	0.0817 (11)	
H3'	−0.0092	−0.2571	0.5883	0.098*	
C4'	0.0689 (4)	−0.2303 (3)	0.53928 (13)	0.0997 (14)	
H4'	0.0612	−0.2974	0.5300	0.120*	
C5'	0.1214 (5)	−0.1580 (3)	0.51803 (12)	0.1061 (15)	
H5'	0.1517	−0.1752	0.4942	0.127*	
C6'	0.1303 (4)	−0.0612 (3)	0.53097 (9)	0.0766 (10)	
H6'	0.1653	−0.0114	0.5156	0.092*	
C1''	0.22222 (19)	0.55868 (17)	0.70302 (6)	0.0365 (5)	
C2''	0.1771 (3)	0.6321 (2)	0.67866 (8)	0.0542 (7)	
C3''	0.1854 (3)	0.7340 (2)	0.68911 (10)	0.0672 (9)	
H3''	0.1571	0.7846	0.6727	0.081*	
C4''	0.2335 (3)	0.7625 (2)	0.72248 (10)	0.0643 (8)	
H4''	0.2361	0.8321	0.7292	0.077*	
C5''	0.2778 (2)	0.6908 (2)	0.74615 (8)	0.0548 (7)	
H5''	0.3122	0.7106	0.7691	0.066*	
C6''	0.2721 (2)	0.59137 (19)	0.73669 (7)	0.0429 (5)	
H6''	0.3029	0.5424	0.7533	0.051*	
N1	−0.0034 (3)	−0.0889 (3)	0.62340 (8)	0.0928 (10)	
H1A	0.0242	−0.0281	0.6312	0.111*	
H1B	0.0201	−0.1384	0.6394	0.111*	
N2	0.01194 (16)	0.38839 (15)	0.54309 (5)	0.0373 (4)	
N3	0.1290 (3)	0.6066 (2)	0.64485 (7)	0.0889 (10)	
H3C	0.0735	0.6526	0.6394	0.107*	
H3D	0.0974	0.5438	0.6467	0.107*	
N4	0.14633 (14)	0.13030 (14)	0.72516 (5)	0.0328 (4)	
O1	0.0612 (2)	0.10686 (18)	0.60764 (5)	0.0740 (6)	
O2	0.14170 (17)	0.13201 (13)	0.55120 (4)	0.0500 (4)	
O3	0.24945 (15)	0.28229 (13)	0.42563 (4)	0.0443 (4)	
H3	0.2200	0.2586	0.4061	0.066*	
O4	0.02651 (16)	0.29539 (14)	0.41009 (5)	0.0487 (4)	
H4	0.0438	0.3130	0.3882	0.073*	
O5	0.17912 (16)	0.43398 (15)	0.37103 (4)	0.0538 (5)	
O6	0.0426 (2)	0.63931 (16)	0.39921 (6)	0.0710 (6)	
O7	0.22516 (17)	0.54113 (14)	0.54284 (5)	0.0554 (5)	
O8	0.16607 (18)	0.41487 (13)	0.66696 (5)	0.0538 (5)	
O9	0.26648 (14)	0.39190 (11)	0.71983 (4)	0.0373 (3)	
O10	0.36612 (14)	−0.01612 (12)	0.71417 (4)	0.0407 (4)	
O11	0.19519 (16)	0.19342 (13)	0.85988 (4)	0.0490 (4)	
H11	0.2113	0.1673	0.8806	0.059*	
O12	0.36556 (16)	0.05052 (14)	0.88956 (4)	0.0481 (4)	
O13	0.22545 (14)	−0.15629 (13)	0.85308 (4)	0.0448 (4)	
O14	0.41392 (15)	0.21944 (12)	0.83770 (4)	0.0441 (4)	
H14A	0.3753	0.2467	0.8599	0.053*	

### Atomic displacement parameters (Å^2^)

**Table:**

	*U*^11^	*U*^22^	*U*^33^	*U*^12^	*U*^13^	*U*^23^
C1	0.0410 (12)	0.0460 (14)	0.0396 (12)	−0.0053 (11)	−0.0029 (10)	−0.0089 (11)
C2	0.0540 (14)	0.0645 (18)	0.0329 (12)	−0.0067 (14)	−0.0057 (11)	−0.0124 (12)
C3	0.0592 (15)	0.0672 (19)	0.0317 (12)	0.0090 (14)	−0.0042 (11)	0.0023 (12)
C4	0.0501 (13)	0.0397 (14)	0.0304 (11)	0.0055 (11)	0.0030 (10)	0.0013 (10)
C5	0.0430 (11)	0.0346 (13)	0.0300 (10)	0.0044 (10)	0.0022 (9)	−0.0028 (9)
C6	0.0528 (13)	0.0309 (12)	0.0328 (11)	−0.0056 (11)	0.0021 (10)	−0.0010 (9)
C7	0.0375 (11)	0.0377 (13)	0.0339 (11)	−0.0049 (10)	0.0020 (9)	−0.0020 (10)
C8	0.0419 (12)	0.0401 (14)	0.0319 (11)	0.0001 (10)	−0.0036 (9)	0.0001 (10)
C9	0.0404 (11)	0.0365 (13)	0.0318 (11)	0.0025 (10)	0.0049 (9)	−0.0010 (9)
C10	0.0363 (11)	0.0398 (13)	0.0346 (11)	−0.0016 (10)	0.0037 (9)	−0.0031 (10)
C11	0.0359 (10)	0.0376 (13)	0.0290 (10)	0.0020 (10)	0.0004 (9)	−0.0028 (9)
C12	0.0649 (16)	0.0497 (16)	0.0476 (14)	−0.0161 (14)	0.0096 (13)	−0.0014 (13)
C13	0.0678 (16)	0.0390 (14)	0.0440 (13)	−0.0073 (13)	0.0152 (12)	0.0097 (11)
C14	0.0482 (12)	0.0507 (15)	0.0373 (12)	0.0017 (12)	0.0070 (10)	0.0046 (11)
C15	0.0514 (13)	0.0521 (16)	0.0403 (13)	0.0120 (12)	0.0034 (11)	0.0064 (12)
C16	0.0776 (18)	0.0401 (14)	0.0415 (13)	0.0126 (14)	0.0174 (13)	0.0076 (11)
C17	0.0403 (11)	0.0304 (12)	0.0302 (10)	0.0013 (9)	0.0027 (9)	−0.0005 (9)
C18	0.0490 (13)	0.0394 (14)	0.0359 (12)	0.0039 (11)	0.0088 (10)	0.0037 (10)
C19	0.0596 (15)	0.0547 (17)	0.0449 (14)	0.0114 (14)	0.0035 (12)	0.0175 (13)
C20	0.107 (3)	0.060 (2)	0.109 (3)	−0.020 (2)	−0.031 (2)	−0.026 (2)
C21	0.126 (3)	0.095 (3)	0.112 (3)	0.070 (3)	0.051 (3)	0.049 (3)
C22	0.096 (2)	0.120 (3)	0.0402 (15)	0.004 (2)	0.0096 (16)	0.0289 (18)
C23	0.0408 (11)	0.0427 (14)	0.0443 (12)	−0.0018 (11)	0.0094 (10)	−0.0046 (11)
C24	0.0607 (16)	0.0464 (17)	0.080 (2)	0.0064 (14)	0.0222 (15)	−0.0036 (15)
C25	0.0344 (10)	0.0266 (11)	0.0352 (11)	−0.0008 (9)	0.0069 (9)	−0.0022 (9)
C26	0.0531 (13)	0.0394 (13)	0.0300 (11)	−0.0009 (11)	0.0092 (10)	−0.0009 (10)
C27	0.0517 (13)	0.0338 (13)	0.0341 (11)	−0.0029 (11)	0.0052 (10)	0.0083 (10)
C28	0.0401 (11)	0.0230 (11)	0.0309 (10)	−0.0018 (9)	−0.0039 (9)	0.0014 (8)
C29	0.0367 (10)	0.0242 (11)	0.0317 (10)	−0.0015 (9)	−0.0028 (9)	−0.0022 (9)
C30	0.0543 (13)	0.0243 (11)	0.0308 (10)	0.0111 (10)	−0.0008 (10)	−0.0002 (9)
C31	0.0354 (10)	0.0297 (12)	0.0351 (11)	0.0092 (9)	0.0042 (9)	0.0005 (9)
C32	0.0416 (11)	0.0325 (12)	0.0309 (10)	0.0075 (10)	0.0079 (9)	0.0010 (9)
C33	0.0409 (11)	0.0282 (12)	0.0324 (11)	−0.0012 (10)	−0.0019 (9)	−0.0016 (9)
C34	0.0307 (9)	0.0291 (12)	0.0338 (11)	0.0006 (9)	0.0024 (8)	0.0009 (9)
C35	0.0304 (9)	0.0233 (11)	0.0278 (10)	−0.0007 (8)	0.0010 (8)	−0.0006 (8)
C36	0.0427 (11)	0.0337 (12)	0.0386 (12)	0.0092 (10)	0.0065 (10)	0.0072 (10)
C37	0.0418 (12)	0.0299 (12)	0.0367 (11)	0.0070 (10)	0.0006 (9)	0.0087 (9)
C38	0.0355 (10)	0.0406 (13)	0.0347 (11)	0.0011 (10)	−0.0011 (9)	0.0046 (10)
C39	0.0363 (10)	0.0428 (14)	0.0382 (12)	0.0029 (10)	0.0061 (9)	0.0104 (10)
C40	0.0452 (12)	0.0309 (12)	0.0337 (11)	−0.0005 (10)	−0.0017 (9)	0.0065 (9)
C41	0.0306 (10)	0.0224 (10)	0.0323 (10)	0.0010 (8)	0.0027 (8)	0.0026 (8)
C42	0.0416 (11)	0.0313 (12)	0.0372 (11)	0.0007 (10)	−0.0066 (9)	0.0037 (10)
C43	0.0458 (12)	0.0311 (12)	0.0335 (11)	−0.0012 (10)	0.0023 (9)	0.0058 (9)
C44	0.098 (2)	0.0424 (16)	0.0711 (19)	0.0198 (17)	0.0277 (18)	−0.0096 (14)
C45	0.0682 (16)	0.0618 (18)	0.0424 (13)	0.0017 (15)	−0.0074 (12)	0.0179 (13)
C46	0.0608 (16)	0.0573 (18)	0.0553 (15)	−0.0205 (15)	−0.0011 (13)	0.0116 (13)
C47	0.0357 (11)	0.0384 (14)	0.0543 (14)	−0.0030 (10)	−0.0076 (10)	0.0064 (11)
C48	0.0518 (15)	0.0461 (17)	0.095 (2)	−0.0143 (13)	−0.0165 (15)	0.0080 (16)
C1'	0.0630 (15)	0.0443 (16)	0.0587 (16)	0.0072 (14)	0.0055 (13)	0.0178 (13)
C2'	0.0569 (15)	0.064 (2)	0.0711 (19)	0.0147 (16)	0.0082 (14)	0.0300 (16)
C3'	0.078 (2)	0.049 (2)	0.118 (3)	−0.0027 (17)	0.004 (2)	0.032 (2)
C4'	0.128 (4)	0.054 (2)	0.117 (3)	−0.021 (2)	0.021 (3)	−0.001 (2)
C5'	0.168 (4)	0.050 (2)	0.101 (3)	−0.022 (3)	0.044 (3)	−0.010 (2)
C6'	0.106 (3)	0.054 (2)	0.070 (2)	−0.010 (2)	0.0229 (19)	0.0025 (16)
C1''	0.0412 (11)	0.0269 (12)	0.0413 (12)	0.0021 (10)	0.0055 (10)	0.0039 (9)
C2''	0.0719 (17)	0.0382 (15)	0.0524 (15)	0.0052 (14)	−0.0059 (13)	0.0087 (12)
C3''	0.089 (2)	0.0286 (14)	0.083 (2)	0.0108 (15)	−0.0110 (18)	0.0108 (14)
C4''	0.0712 (18)	0.0291 (14)	0.093 (2)	0.0004 (14)	−0.0004 (17)	−0.0093 (15)
C5''	0.0589 (15)	0.0367 (14)	0.0689 (17)	−0.0007 (13)	−0.0072 (14)	−0.0109 (13)
C6''	0.0450 (12)	0.0373 (14)	0.0463 (13)	0.0005 (11)	0.0011 (10)	−0.0011 (11)
N1	0.115 (2)	0.087 (2)	0.0763 (19)	0.014 (2)	0.0178 (18)	0.0409 (18)
N2	0.0414 (10)	0.0371 (11)	0.0335 (9)	0.0000 (9)	0.0076 (8)	−0.0006 (8)
N3	0.145 (3)	0.0551 (16)	0.0664 (16)	0.0170 (19)	−0.0368 (18)	0.0142 (14)
N4	0.0334 (8)	0.0272 (10)	0.0378 (9)	−0.0026 (8)	−0.0044 (7)	0.0050 (8)
O1	0.1100 (17)	0.0679 (14)	0.0440 (10)	0.0173 (14)	0.0202 (11)	0.0133 (10)
O2	0.0732 (11)	0.0378 (10)	0.0391 (9)	0.0081 (9)	0.0077 (8)	0.0060 (7)
O3	0.0529 (9)	0.0415 (10)	0.0384 (8)	0.0108 (8)	0.0026 (7)	−0.0044 (7)
O4	0.0586 (10)	0.0535 (11)	0.0339 (8)	−0.0027 (9)	−0.0060 (8)	−0.0057 (8)
O5	0.0633 (11)	0.0652 (12)	0.0329 (8)	0.0044 (10)	0.0075 (8)	0.0056 (8)
O6	0.0880 (14)	0.0576 (13)	0.0673 (12)	0.0311 (12)	0.0275 (11)	0.0269 (10)
O7	0.0685 (11)	0.0434 (11)	0.0543 (10)	−0.0052 (9)	−0.0096 (9)	−0.0171 (9)
O8	0.0842 (13)	0.0365 (9)	0.0406 (9)	0.0002 (9)	−0.0173 (9)	0.0018 (8)
O9	0.0536 (9)	0.0210 (7)	0.0374 (8)	0.0001 (7)	−0.0058 (7)	0.0033 (6)
O10	0.0546 (9)	0.0263 (8)	0.0411 (8)	0.0031 (7)	0.0062 (8)	−0.0068 (7)
O11	0.0695 (11)	0.0457 (10)	0.0319 (8)	0.0154 (9)	0.0094 (8)	−0.0014 (7)
O12	0.0626 (10)	0.0508 (11)	0.0309 (8)	0.0075 (9)	−0.0024 (7)	0.0074 (7)
O13	0.0534 (9)	0.0359 (9)	0.0452 (9)	−0.0099 (8)	−0.0054 (8)	0.0136 (7)
O14	0.0593 (10)	0.0344 (9)	0.0387 (8)	−0.0057 (8)	−0.0036 (8)	−0.0029 (7)

### Geometric parameters (Å, º)

**Table:**

C1—O7	1.431 (3)	C29—H29	1.0000
C1—C2	1.530 (3)	C30—C31	1.532 (3)
C1—C11	1.549 (3)	C30—H30A	0.9900
C1—H1	1.0000	C30—H30B	0.9900
C2—C3	1.512 (4)	C31—C32	1.534 (3)
C2—H2A	0.9900	C31—C41	1.556 (3)
C2—H2B	0.9900	C31—H31	1.0000
C3—C4	1.504 (3)	C32—O11	1.450 (3)
C3—H3A	0.9900	C32—C39	1.546 (3)
C3—H3B	0.9900	C32—C33	1.576 (3)
C4—O2	1.467 (3)	C33—O14	1.419 (3)
C4—C5	1.538 (3)	C33—C38	1.526 (3)
C4—C18	1.539 (3)	C33—C34	1.554 (3)
C5—C6	1.548 (3)	C34—C36	1.564 (3)
C5—C11	1.552 (3)	C34—C35	1.566 (3)
C5—H5	1.0000	C34—H34	1.0000
C6—C7	1.534 (3)	C35—C41	1.535 (3)
C6—H6A	0.9900	C36—C37	1.543 (3)
C6—H6B	0.9900	C36—H36A	0.9900
C7—C8	1.532 (3)	C36—H36B	0.9900
C7—C17	1.544 (3)	C37—C38	1.514 (3)
C7—H7	1.0000	C37—C40	1.536 (3)
C8—O4	1.441 (3)	C37—H37	1.0000
C8—C15	1.546 (3)	C38—O12	1.429 (3)
C8—C9	1.569 (3)	C38—H38	1.0000
C9—O3	1.421 (3)	C39—C40	1.536 (3)
C9—C14	1.511 (3)	C39—H39A	0.9900
C9—C10	1.551 (3)	C39—H39B	0.9900
C10—C12	1.565 (3)	C40—O13	1.432 (3)
C10—C11	1.565 (3)	C40—H40	1.0000
C10—H10	1.0000	C41—N4	1.470 (3)
C11—C17	1.536 (3)	C41—H41	1.0000
C12—C13	1.543 (4)	C42—N4	1.466 (3)
C12—H12A	0.9900	C42—H42A	0.9900
C12—H12B	0.9900	C42—H42B	0.9900
C13—C14	1.513 (4)	C43—O8	1.218 (3)
C13—C16	1.530 (4)	C43—O9	1.346 (3)
C13—H13	1.0000	C43—C1''	1.467 (3)
C14—O5	1.427 (3)	C44—O10	1.425 (3)
C14—H14	1.0000	C44—H44A	0.9800
C15—C16	1.526 (4)	C44—H44B	0.9800
C15—H15A	0.9900	C44—H44C	0.9800
C15—H15B	0.9900	C45—O12	1.415 (3)
C16—O6	1.429 (3)	C45—H45A	0.9800
C16—H16	1.0000	C45—H45B	0.9800
C17—N2	1.474 (3)	C45—H45C	0.9800
C17—H17	1.0000	C46—O13	1.411 (3)
C18—N2	1.462 (3)	C46—H46A	0.9800
C18—H18A	0.9900	C46—H46B	0.9800
C18—H18B	0.9900	C46—H46C	0.9800
C19—O1	1.217 (3)	C47—N4	1.459 (3)
C19—O2	1.343 (3)	C47—C48	1.507 (4)
C19—C1'	1.478 (4)	C47—H47A	0.9900
C20—O7	1.409 (4)	C47—H47B	0.9900
C20—H20A	0.9800	C48—H48A	0.9800
C20—H20B	0.9800	C48—H48B	0.9800
C20—H20C	0.9800	C48—H48C	0.9800
C21—O6	1.419 (4)	C1'—C6'	1.390 (4)
C21—H21A	0.9800	C1'—C2'	1.408 (4)
C21—H21B	0.9800	C2'—N1	1.368 (4)
C21—H21C	0.9800	C2'—C3'	1.395 (5)
C22—O5	1.403 (3)	C3'—C4'	1.364 (6)
C22—H22A	0.9800	C3'—H3'	0.9500
C22—H22B	0.9800	C4'—C5'	1.366 (5)
C22—H22C	0.9800	C4'—H4'	0.9500
C23—N2	1.460 (3)	C5'—C6'	1.363 (5)
C23—C24	1.508 (4)	C5'—H5'	0.9500
C23—H23A	0.9900	C6'—H6'	0.9500
C23—H23B	0.9900	C1''—C6''	1.405 (3)
C24—H24A	0.9800	C1''—C2''	1.406 (3)
C24—H24B	0.9800	C2''—N3	1.375 (4)
C24—H24C	0.9800	C2''—C3''	1.400 (4)
C25—O10	1.421 (3)	C3''—C4''	1.372 (4)
C25—C26	1.535 (3)	C3''—H3''	0.9500
C25—C35	1.555 (3)	C4''—C5''	1.372 (4)
C25—H25	1.0000	C4''—H4''	0.9500
C26—C27	1.508 (3)	C5''—C6''	1.357 (3)
C26—H26A	0.9900	C5''—H5''	0.9500
C26—H26B	0.9900	C6''—H6''	0.9500
C27—C28	1.519 (3)	N1—H1A	0.9100
C27—H27A	0.9900	N1—H1B	0.9100
C27—H27B	0.9900	N3—H3C	0.9100
C28—O9	1.467 (2)	N3—H3D	0.9100
C28—C29	1.528 (3)	O3—H3	0.8400
C28—C42	1.543 (3)	O4—H4	0.8400
C29—C30	1.551 (3)	O11—H11	0.8400
C29—C35	1.555 (3)	O14—H14A	0.9800
			
O7—C1—C2	107.8 (2)	C31—C30—C29	105.27 (17)
O7—C1—C11	108.43 (19)	C31—C30—H30A	110.7
C2—C1—C11	117.1 (2)	C29—C30—H30A	110.7
O7—C1—H1	107.7	C31—C30—H30B	110.7
C2—C1—H1	107.7	C29—C30—H30B	110.7
C11—C1—H1	107.7	H30A—C30—H30B	108.8
C3—C2—C1	113.8 (2)	C30—C31—C32	111.15 (18)
C3—C2—H2A	108.8	C30—C31—C41	102.96 (16)
C1—C2—H2A	108.8	C32—C31—C41	110.98 (16)
C3—C2—H2B	108.8	C30—C31—H31	110.5
C1—C2—H2B	108.8	C32—C31—H31	110.5
H2A—C2—H2B	107.7	C41—C31—H31	110.5
C4—C3—C2	109.3 (2)	O11—C32—C31	105.12 (17)
C4—C3—H3A	109.8	O11—C32—C39	107.97 (17)
C2—C3—H3A	109.8	C31—C32—C39	112.04 (19)
C4—C3—H3B	109.8	O11—C32—C33	108.49 (19)
C2—C3—H3B	109.8	C31—C32—C33	110.21 (17)
H3A—C3—H3B	108.3	C39—C32—C33	112.65 (18)
O2—C4—C3	109.8 (2)	O14—C33—C38	111.30 (17)
O2—C4—C5	101.74 (18)	O14—C33—C34	108.18 (17)
C3—C4—C5	110.8 (2)	C38—C33—C34	102.86 (17)
O2—C4—C18	110.4 (2)	O14—C33—C32	113.09 (19)
C3—C4—C18	114.4 (2)	C38—C33—C32	109.54 (18)
C5—C4—C18	108.98 (19)	C34—C33—C32	111.41 (17)
C4—C5—C6	108.03 (19)	C33—C34—C36	103.19 (17)
C4—C5—C11	108.83 (18)	C33—C34—C35	117.85 (16)
C6—C5—C11	105.32 (17)	C36—C34—C35	116.19 (18)
C4—C5—H5	111.5	C33—C34—H34	106.2
C6—C5—H5	111.5	C36—C34—H34	106.2
C11—C5—H5	111.5	C35—C34—H34	106.2
C7—C6—C5	104.39 (17)	C41—C35—C29	98.14 (16)
C7—C6—H6A	110.9	C41—C35—C25	116.01 (17)
C5—C6—H6A	110.9	C29—C35—C25	112.82 (16)
C7—C6—H6B	110.9	C41—C35—C34	110.27 (16)
C5—C6—H6B	110.9	C29—C35—C34	111.05 (16)
H6A—C6—H6B	108.9	C25—C35—C34	108.30 (16)
C8—C7—C6	110.69 (18)	C37—C36—C34	106.70 (18)
C8—C7—C17	111.72 (18)	C37—C36—H36A	110.4
C6—C7—C17	103.77 (17)	C34—C36—H36A	110.4
C8—C7—H7	110.2	C37—C36—H36B	110.4
C6—C7—H7	110.2	C34—C36—H36B	110.4
C17—C7—H7	110.2	H36A—C36—H36B	108.6
O4—C8—C7	105.52 (18)	C38—C37—C40	113.19 (19)
O4—C8—C15	108.17 (18)	C38—C37—C36	99.25 (18)
C7—C8—C15	111.87 (19)	C40—C37—C36	110.85 (18)
O4—C8—C9	108.27 (19)	C38—C37—H37	111.0
C7—C8—C9	109.72 (18)	C40—C37—H37	111.0
C15—C8—C9	112.9 (2)	C36—C37—H37	111.0
O3—C9—C14	110.43 (18)	O12—C38—C37	118.60 (19)
O3—C9—C10	108.94 (18)	O12—C38—C33	109.01 (18)
C14—C9—C10	102.78 (19)	C37—C38—C33	102.50 (17)
O3—C9—C8	112.8 (2)	O12—C38—H38	108.8
C14—C9—C8	109.8 (2)	C37—C38—H38	108.8
C10—C9—C8	111.68 (17)	C33—C38—H38	108.8
C9—C10—C12	103.03 (19)	C40—C39—C32	118.16 (18)
C9—C10—C11	117.51 (18)	C40—C39—H39A	107.8
C12—C10—C11	115.81 (19)	C32—C39—H39A	107.8
C9—C10—H10	106.6	C40—C39—H39B	107.8
C12—C10—H10	106.6	C32—C39—H39B	107.8
C11—C10—H10	106.6	H39A—C39—H39B	107.1
C17—C11—C1	116.23 (18)	O13—C40—C39	110.64 (18)
C17—C11—C5	98.06 (18)	O13—C40—C37	108.11 (18)
C1—C11—C5	112.57 (18)	C39—C40—C37	113.07 (19)
C17—C11—C10	109.21 (17)	O13—C40—H40	108.3
C1—C11—C10	108.58 (18)	C39—C40—H40	108.3
C5—C11—C10	111.92 (18)	C37—C40—H40	108.3
C13—C12—C10	106.9 (2)	N4—C41—C35	108.80 (16)
C13—C12—H12A	110.3	N4—C41—C31	115.46 (16)
C10—C12—H12A	110.3	C35—C41—C31	100.84 (16)
C13—C12—H12B	110.3	N4—C41—H41	110.4
C10—C12—H12B	110.3	C35—C41—H41	110.4
H12A—C12—H12B	108.6	C31—C41—H41	110.4
C14—C13—C16	112.5 (2)	N4—C42—C28	113.15 (17)
C14—C13—C12	99.8 (2)	N4—C42—H42A	108.9
C16—C13—C12	111.2 (2)	C28—C42—H42A	108.9
C14—C13—H13	111.0	N4—C42—H42B	108.9
C16—C13—H13	111.0	C28—C42—H42B	108.9
C12—C13—H13	111.0	H42A—C42—H42B	107.8
O5—C14—C9	108.6 (2)	O8—C43—O9	122.2 (2)
O5—C14—C13	117.9 (2)	O8—C43—C1''	125.3 (2)
C9—C14—C13	102.86 (19)	O9—C43—C1''	112.53 (19)
O5—C14—H14	109.0	O10—C44—H44A	109.5
C9—C14—H14	109.0	O10—C44—H44B	109.5
C13—C14—H14	109.0	H44A—C44—H44B	109.5
C16—C15—C8	119.1 (2)	O10—C44—H44C	109.5
C16—C15—H15A	107.5	H44A—C44—H44C	109.5
C8—C15—H15A	107.5	H44B—C44—H44C	109.5
C16—C15—H15B	107.5	O12—C45—H45A	109.5
C8—C15—H15B	107.5	O12—C45—H45B	109.5
H15A—C15—H15B	107.0	H45A—C45—H45B	109.5
O6—C16—C15	111.0 (2)	O12—C45—H45C	109.5
O6—C16—C13	107.2 (2)	H45A—C45—H45C	109.5
C15—C16—C13	113.5 (2)	H45B—C45—H45C	109.5
O6—C16—H16	108.3	O13—C46—H46A	109.5
C15—C16—H16	108.3	O13—C46—H46B	109.5
C13—C16—H16	108.3	H46A—C46—H46B	109.5
N2—C17—C11	108.60 (17)	O13—C46—H46C	109.5
N2—C17—C7	115.47 (18)	H46A—C46—H46C	109.5
C11—C17—C7	100.71 (17)	H46B—C46—H46C	109.5
N2—C17—H17	110.5	N4—C47—C48	114.3 (2)
C11—C17—H17	110.5	N4—C47—H47A	108.7
C7—C17—H17	110.5	C48—C47—H47A	108.7
N2—C18—C4	112.4 (2)	N4—C47—H47B	108.7
N2—C18—H18A	109.1	C48—C47—H47B	108.7
C4—C18—H18A	109.1	H47A—C47—H47B	107.6
N2—C18—H18B	109.1	C47—C48—H48A	109.5
C4—C18—H18B	109.1	C47—C48—H48B	109.5
H18A—C18—H18B	107.8	H48A—C48—H48B	109.5
O1—C19—O2	123.0 (3)	C47—C48—H48C	109.5
O1—C19—C1'	125.6 (3)	H48A—C48—H48C	109.5
O2—C19—C1'	111.4 (2)	H48B—C48—H48C	109.5
O7—C20—H20A	109.5	C6'—C1'—C2'	118.2 (3)
O7—C20—H20B	109.5	C6'—C1'—C19	119.7 (3)
H20A—C20—H20B	109.5	C2'—C1'—C19	122.0 (3)
O7—C20—H20C	109.5	N1—C2'—C3'	119.0 (3)
H20A—C20—H20C	109.5	N1—C2'—C1'	122.4 (3)
H20B—C20—H20C	109.5	C3'—C2'—C1'	118.6 (3)
O6—C21—H21A	109.5	C4'—C3'—C2'	121.3 (3)
O6—C21—H21B	109.5	C4'—C3'—H3'	119.3
H21A—C21—H21B	109.5	C2'—C3'—H3'	119.3
O6—C21—H21C	109.5	C3'—C4'—C5'	120.0 (4)
H21A—C21—H21C	109.5	C3'—C4'—H4'	120.0
H21B—C21—H21C	109.5	C5'—C4'—H4'	120.0
O5—C22—H22A	109.5	C6'—C5'—C4'	120.1 (4)
O5—C22—H22B	109.5	C6'—C5'—H5'	120.0
H22A—C22—H22B	109.5	C4'—C5'—H5'	120.0
O5—C22—H22C	109.5	C5'—C6'—C1'	121.7 (3)
H22A—C22—H22C	109.5	C5'—C6'—H6'	119.1
H22B—C22—H22C	109.5	C1'—C6'—H6'	119.1
N2—C23—C24	113.5 (2)	C6''—C1''—C2''	118.3 (2)
N2—C23—H23A	108.9	C6''—C1''—C43	120.7 (2)
C24—C23—H23A	108.9	C2''—C1''—C43	120.9 (2)
N2—C23—H23B	108.9	N3—C2''—C3''	119.9 (3)
C24—C23—H23B	108.9	N3—C2''—C1''	122.0 (3)
H23A—C23—H23B	107.7	C3''—C2''—C1''	118.1 (3)
C23—C24—H24A	109.5	C4''—C3''—C2''	121.7 (3)
C23—C24—H24B	109.5	C4''—C3''—H3''	119.2
H24A—C24—H24B	109.5	C2''—C3''—H3''	119.2
C23—C24—H24C	109.5	C3''—C4''—C5''	120.1 (3)
H24A—C24—H24C	109.5	C3''—C4''—H4''	120.0
H24B—C24—H24C	109.5	C5''—C4''—H4''	120.0
O10—C25—C26	108.00 (17)	C6''—C5''—C4''	119.7 (3)
O10—C25—C35	109.78 (16)	C6''—C5''—H5''	120.1
C26—C25—C35	116.81 (17)	C4''—C5''—H5''	120.1
O10—C25—H25	107.3	C5''—C6''—C1''	122.1 (2)
C26—C25—H25	107.3	C5''—C6''—H6''	119.0
C35—C25—H25	107.3	C1''—C6''—H6''	119.0
C27—C26—C25	113.19 (19)	C2'—N1—H1A	108.9
C27—C26—H26A	108.9	C2'—N1—H1B	109.3
C25—C26—H26A	108.9	H1A—N1—H1B	109.5
C27—C26—H26B	108.9	C23—N2—C18	111.77 (19)
C25—C26—H26B	108.9	C23—N2—C17	114.02 (19)
H26A—C26—H26B	107.8	C18—N2—C17	116.80 (18)
C26—C27—C28	109.07 (18)	C2''—N3—H3C	108.5
C26—C27—H27A	109.9	C2''—N3—H3D	109.0
C28—C27—H27A	109.9	H3C—N3—H3D	109.5
C26—C27—H27B	109.9	C47—N4—C42	109.67 (17)
C28—C27—H27B	109.9	C47—N4—C41	114.24 (17)
H27A—C27—H27B	108.3	C42—N4—C41	117.19 (17)
O9—C28—C27	109.62 (17)	C19—O2—C4	124.4 (2)
O9—C28—C29	102.89 (16)	C9—O3—H3	109.5
C27—C28—C29	110.14 (18)	C8—O4—H4	109.5
O9—C28—C42	110.62 (17)	C22—O5—C14	113.9 (2)
C27—C28—C42	114.03 (18)	C21—O6—C16	112.6 (2)
C29—C28—C42	108.97 (17)	C20—O7—C1	114.2 (2)
C28—C29—C30	108.75 (17)	C43—O9—C28	122.13 (17)
C28—C29—C35	109.14 (17)	C25—O10—C44	113.1 (2)
C30—C29—C35	104.76 (17)	C32—O11—H11	109.4
C28—C29—H29	111.3	C45—O12—C38	112.77 (19)
C30—C29—H29	111.3	C46—O13—C40	112.65 (18)
C35—C29—H29	111.3	C33—O14—H14A	109.3
			
O7—C1—C2—C3	161.3 (2)	O14—C33—C34—C35	−89.7 (2)
C11—C1—C2—C3	38.9 (3)	C38—C33—C34—C35	152.49 (18)
C1—C2—C3—C4	−51.2 (3)	C32—C33—C34—C35	35.2 (3)
C2—C3—C4—O2	177.0 (2)	C28—C29—C35—C41	−74.90 (19)
C2—C3—C4—C5	65.4 (3)	C30—C29—C35—C41	41.42 (19)
C2—C3—C4—C18	−58.2 (3)	C28—C29—C35—C25	47.8 (2)
O2—C4—C5—C6	65.3 (2)	C30—C29—C35—C25	164.15 (17)
C3—C4—C5—C6	−178.0 (2)	C28—C29—C35—C34	169.64 (16)
C18—C4—C5—C6	−51.3 (2)	C30—C29—C35—C34	−74.0 (2)
O2—C4—C5—C11	179.17 (18)	O10—C25—C35—C41	−48.4 (2)
C3—C4—C5—C11	−64.2 (3)	C26—C25—C35—C41	74.9 (2)
C18—C4—C5—C11	62.5 (2)	O10—C25—C35—C29	−160.53 (17)
C4—C5—C6—C7	101.3 (2)	C26—C25—C35—C29	−37.2 (3)
C11—C5—C6—C7	−14.9 (2)	O10—C25—C35—C34	76.1 (2)
C5—C6—C7—C8	102.43 (19)	C26—C25—C35—C34	−160.53 (19)
C5—C6—C7—C17	−17.6 (2)	C33—C34—C35—C41	−49.7 (2)
C6—C7—C8—O4	64.9 (2)	C36—C34—C35—C41	73.5 (2)
C17—C7—C8—O4	179.98 (18)	C33—C34—C35—C29	58.0 (2)
C6—C7—C8—C15	−177.73 (19)	C36—C34—C35—C29	−178.83 (17)
C17—C7—C8—C15	−62.6 (2)	C33—C34—C35—C25	−177.63 (18)
C6—C7—C8—C9	−51.6 (2)	C36—C34—C35—C25	−54.4 (2)
C17—C7—C8—C9	63.5 (2)	C33—C34—C36—C37	7.3 (2)
O4—C8—C9—O3	−31.9 (2)	C35—C34—C36—C37	−123.20 (19)
C7—C8—C9—O3	82.8 (2)	C34—C36—C37—C38	−34.7 (2)
C15—C8—C9—O3	−151.70 (19)	C34—C36—C37—C40	84.5 (2)
O4—C8—C9—C14	91.7 (2)	C40—C37—C38—O12	52.1 (3)
C7—C8—C9—C14	−153.64 (19)	C36—C37—C38—O12	169.60 (18)
C15—C8—C9—C14	−28.1 (3)	C40—C37—C38—C33	−68.0 (2)
O4—C8—C9—C10	−155.01 (18)	C36—C37—C38—C33	49.5 (2)
C7—C8—C9—C10	−40.3 (3)	O14—C33—C38—O12	71.7 (2)
C15—C8—C9—C10	85.2 (2)	C34—C33—C38—O12	−172.71 (17)
O3—C9—C10—C12	143.00 (19)	C32—C33—C38—O12	−54.1 (2)
C14—C9—C10—C12	25.9 (2)	O14—C33—C38—C37	−161.82 (18)
C8—C9—C10—C12	−91.8 (2)	C34—C33—C38—C37	−46.2 (2)
O3—C9—C10—C11	−88.4 (2)	C32—C33—C38—C37	72.4 (2)
C14—C9—C10—C11	154.5 (2)	O11—C32—C39—C40	−143.67 (19)
C8—C9—C10—C11	36.9 (3)	C31—C32—C39—C40	101.0 (2)
O7—C1—C11—C17	−47.8 (3)	C33—C32—C39—C40	−23.9 (3)
C2—C1—C11—C17	74.3 (3)	C32—C39—C40—O13	150.93 (19)
O7—C1—C11—C5	−159.75 (19)	C32—C39—C40—C37	29.5 (3)
C2—C1—C11—C5	−37.6 (3)	C38—C37—C40—O13	−105.1 (2)
O7—C1—C11—C10	75.8 (2)	C36—C37—C40—O13	144.42 (19)
C2—C1—C11—C10	−162.1 (2)	C38—C37—C40—C39	17.8 (3)
C4—C5—C11—C17	−74.4 (2)	C36—C37—C40—C39	−92.7 (2)
C6—C5—C11—C17	41.2 (2)	C29—C35—C41—N4	69.81 (19)
C4—C5—C11—C1	48.4 (2)	C25—C35—C41—N4	−50.6 (2)
C6—C5—C11—C1	164.07 (18)	C34—C35—C41—N4	−174.11 (16)
C4—C5—C11—C10	171.08 (18)	C29—C35—C41—C31	−52.03 (17)
C6—C5—C11—C10	−73.3 (2)	C25—C35—C41—C31	−172.41 (16)
C9—C10—C11—C17	−51.6 (3)	C34—C35—C41—C31	64.05 (19)
C12—C10—C11—C17	70.7 (3)	C30—C31—C41—N4	−73.1 (2)
C9—C10—C11—C1	−179.26 (19)	C32—C31—C41—N4	167.96 (17)
C12—C10—C11—C1	−57.0 (3)	C30—C31—C41—C35	43.99 (19)
C9—C10—C11—C5	55.9 (3)	C32—C31—C41—C35	−75.0 (2)
C12—C10—C11—C5	178.2 (2)	O9—C28—C42—N4	−153.09 (17)
C9—C10—C12—C13	3.5 (2)	C27—C28—C42—N4	82.8 (2)
C11—C10—C12—C13	−126.2 (2)	C29—C28—C42—N4	−40.7 (2)
C10—C12—C13—C14	−31.2 (3)	O1—C19—C1'—C6'	179.2 (3)
C10—C12—C13—C16	87.7 (3)	O2—C19—C1'—C6'	−0.1 (4)
O3—C9—C14—O5	71.1 (2)	O1—C19—C1'—C2'	3.2 (5)
C10—C9—C14—O5	−172.86 (18)	O2—C19—C1'—C2'	−176.2 (3)
C8—C9—C14—O5	−53.9 (3)	C6'—C1'—C2'—N1	179.3 (3)
O3—C9—C14—C13	−163.2 (2)	C19—C1'—C2'—N1	−4.6 (5)
C10—C9—C14—C13	−47.2 (2)	C6'—C1'—C2'—C3'	−1.6 (4)
C8—C9—C14—C13	71.8 (2)	C19—C1'—C2'—C3'	174.5 (3)
C16—C13—C14—O5	49.6 (3)	N1—C2'—C3'—C4'	−179.0 (4)
C12—C13—C14—O5	167.5 (2)	C1'—C2'—C3'—C4'	1.9 (5)
C16—C13—C14—C9	−69.9 (3)	C2'—C3'—C4'—C5'	−0.3 (7)
C12—C13—C14—C9	48.1 (2)	C3'—C4'—C5'—C6'	−1.4 (8)
O4—C8—C15—C16	−140.2 (2)	C4'—C5'—C6'—C1'	1.6 (8)
C7—C8—C15—C16	104.0 (2)	C2'—C1'—C6'—C5'	−0.1 (6)
C9—C8—C15—C16	−20.4 (3)	C19—C1'—C6'—C5'	−176.3 (4)
C8—C15—C16—O6	144.3 (2)	O8—C43—C1''—C6''	174.7 (2)
C8—C15—C16—C13	23.5 (3)	O9—C43—C1''—C6''	−4.4 (3)
C14—C13—C16—O6	−100.5 (3)	O8—C43—C1''—C2''	−3.5 (4)
C12—C13—C16—O6	148.5 (2)	O9—C43—C1''—C2''	177.4 (2)
C14—C13—C16—C15	22.4 (3)	C6''—C1''—C2''—N3	178.6 (3)
C12—C13—C16—C15	−88.6 (3)	C43—C1''—C2''—N3	−3.1 (4)
C1—C11—C17—N2	−50.0 (3)	C6''—C1''—C2''—C3''	0.6 (4)
C5—C11—C17—N2	70.1 (2)	C43—C1''—C2''—C3''	178.9 (3)
C10—C11—C17—N2	−173.27 (17)	N3—C2''—C3''—C4''	−179.6 (3)
C1—C11—C17—C7	−171.74 (19)	C1''—C2''—C3''—C4''	−1.6 (5)
C5—C11—C17—C7	−51.62 (19)	C2''—C3''—C4''—C5''	1.8 (5)
C10—C11—C17—C7	65.0 (2)	C3''—C4''—C5''—C6''	−1.1 (5)
C8—C7—C17—N2	167.96 (18)	C4''—C5''—C6''—C1''	0.2 (4)
C6—C7—C17—N2	−72.8 (2)	C2''—C1''—C6''—C5''	0.0 (4)
C8—C7—C17—C11	−75.3 (2)	C43—C1''—C6''—C5''	−178.3 (2)
C6—C7—C17—C11	44.0 (2)	C24—C23—N2—C18	157.0 (2)
O2—C4—C18—N2	−153.59 (18)	C24—C23—N2—C17	−67.8 (3)
C3—C4—C18—N2	82.0 (3)	C4—C18—N2—C23	176.49 (19)
C5—C4—C18—N2	−42.6 (3)	C4—C18—N2—C17	42.6 (3)
O10—C25—C26—C27	164.22 (19)	C11—C17—N2—C23	168.01 (19)
C35—C25—C26—C27	40.0 (3)	C7—C17—N2—C23	−79.8 (3)
C25—C26—C27—C28	−53.2 (3)	C11—C17—N2—C18	−59.1 (2)
C26—C27—C28—O9	179.43 (18)	C7—C17—N2—C18	53.0 (3)
C26—C27—C28—C29	66.9 (2)	C48—C47—N4—C42	165.8 (2)
C26—C27—C28—C42	−55.9 (3)	C48—C47—N4—C41	−60.4 (3)
O9—C28—C29—C30	65.5 (2)	C28—C42—N4—C47	172.51 (19)
C27—C28—C29—C30	−177.69 (18)	C28—C42—N4—C41	40.2 (2)
C42—C28—C29—C30	−51.9 (2)	C35—C41—N4—C47	172.30 (19)
O9—C28—C29—C35	179.26 (16)	C31—C41—N4—C47	−75.2 (2)
C27—C28—C29—C35	−63.9 (2)	C35—C41—N4—C42	−57.4 (2)
C42—C28—C29—C35	61.8 (2)	C31—C41—N4—C42	55.0 (2)
C28—C29—C30—C31	101.63 (19)	O1—C19—O2—C4	−7.9 (4)
C35—C29—C30—C31	−15.0 (2)	C1'—C19—O2—C4	171.5 (2)
C29—C30—C31—C32	101.46 (19)	C3—C4—O2—C19	73.0 (3)
C29—C30—C31—C41	−17.4 (2)	C5—C4—O2—C19	−169.6 (2)
C30—C31—C32—O11	67.1 (2)	C18—C4—O2—C19	−54.0 (3)
C41—C31—C32—O11	−179.00 (17)	C9—C14—O5—C22	−160.9 (3)
C30—C31—C32—C39	−175.93 (17)	C13—C14—O5—C22	82.7 (3)
C41—C31—C32—C39	−62.0 (2)	C15—C16—O6—C21	68.2 (4)
C30—C31—C32—C33	−49.7 (2)	C13—C16—O6—C21	−167.3 (3)
C41—C31—C32—C33	64.3 (2)	C2—C1—O7—C20	81.8 (3)
O11—C32—C33—O14	−33.1 (2)	C11—C1—O7—C20	−150.6 (3)
C31—C32—C33—O14	81.5 (2)	O8—C43—O9—C28	3.2 (3)
C39—C32—C33—O14	−152.54 (18)	C1''—C43—O9—C28	−177.64 (18)
O11—C32—C33—C38	91.7 (2)	C27—C28—O9—C43	70.0 (2)
C31—C32—C33—C38	−153.70 (18)	C29—C28—O9—C43	−172.83 (18)
C39—C32—C33—C38	−27.8 (2)	C42—C28—O9—C43	−56.6 (2)
O11—C32—C33—C34	−155.17 (17)	C26—C25—O10—C44	79.8 (2)
C31—C32—C33—C34	−40.6 (2)	C35—C25—O10—C44	−151.8 (2)
C39—C32—C33—C34	85.4 (2)	C37—C38—O12—C45	64.2 (3)
O14—C33—C34—C36	140.79 (18)	C33—C38—O12—C45	−179.2 (2)
C38—C33—C34—C36	22.9 (2)	C39—C40—O13—C46	75.9 (3)
C32—C33—C34—C36	−94.3 (2)	C37—C40—O13—C46	−159.8 (2)

### Hydrogen-bond geometry (Å, º)

**Table:**

*D*—H···*A*	*D*—H	H···*A*	*D*···*A*	*D*—H···*A*
O14—H14*A*···O6^i^	0.98	2.27	2.927 (2)	123
O11—H11···O12	0.84	2.40	2.944 (2)	124
O4—H4···O5	0.84	2.33	2.914 (3)	127
O3—H3···O13^ii^	0.84	2.41	3.095 (2)	139
N3—H3*D*···O8	0.91	2.02	2.687 (3)	129
N1—H1*A*···O1	0.91	2.02	2.752 (4)	137

Symmetry codes: (i) −*x*+1/2, −*y*+1, *z*+1/2; (ii) −*x*+1/2, −*y*, *z*−1/2.
