# Supplementary figures and images for: Crystal structure of N-de­acetyl­lappa­coni­tine
Source: Acta Crystallogr E Crystallogr Commun. 2015 Jul 15;71(Pt 8):o576–7. doi: 10.1107/S2056989015012335 (PMC4571405; doi:10.1107/S2056989015012335)

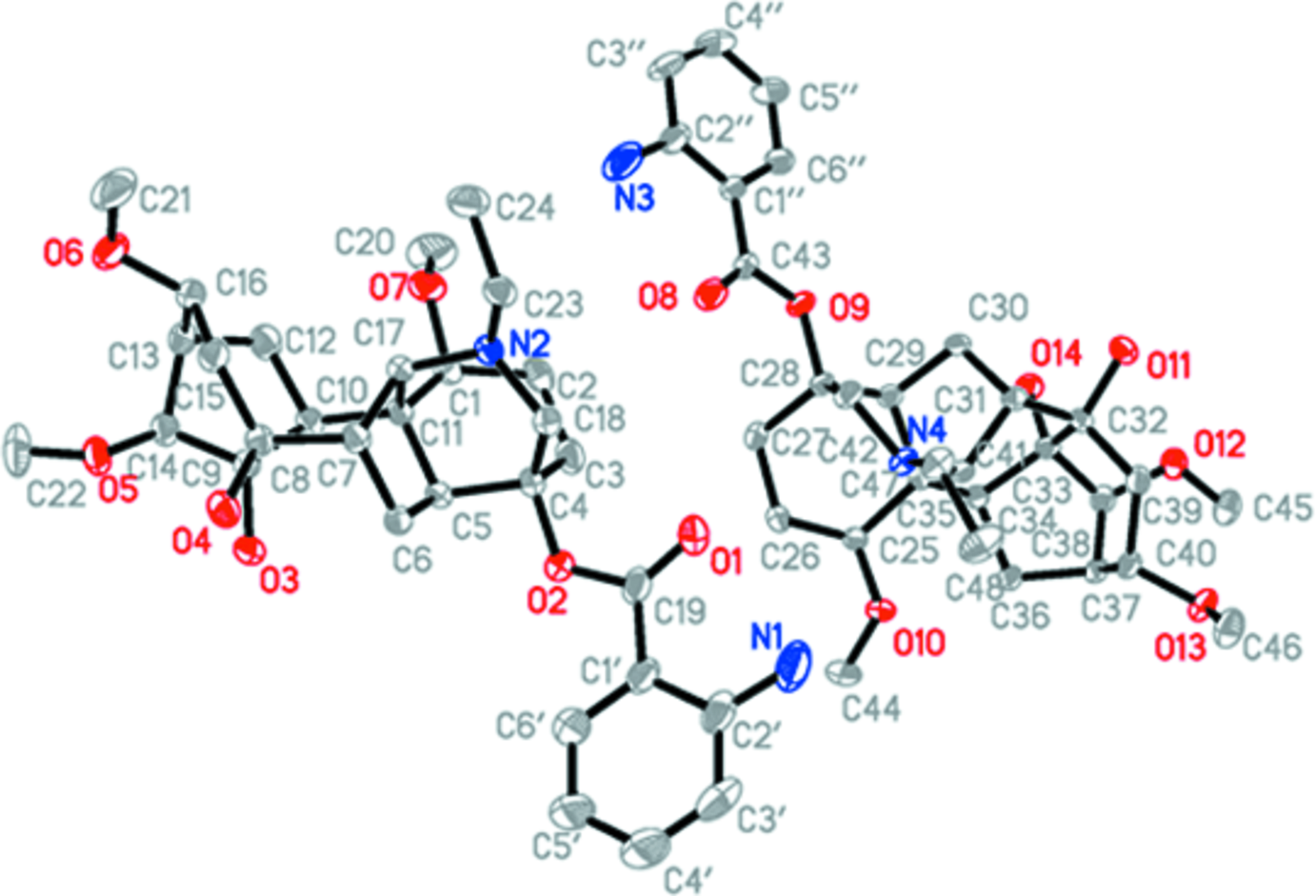

Supplement: Supplementary file 4 [file e-71-0o576-fig1.tif]

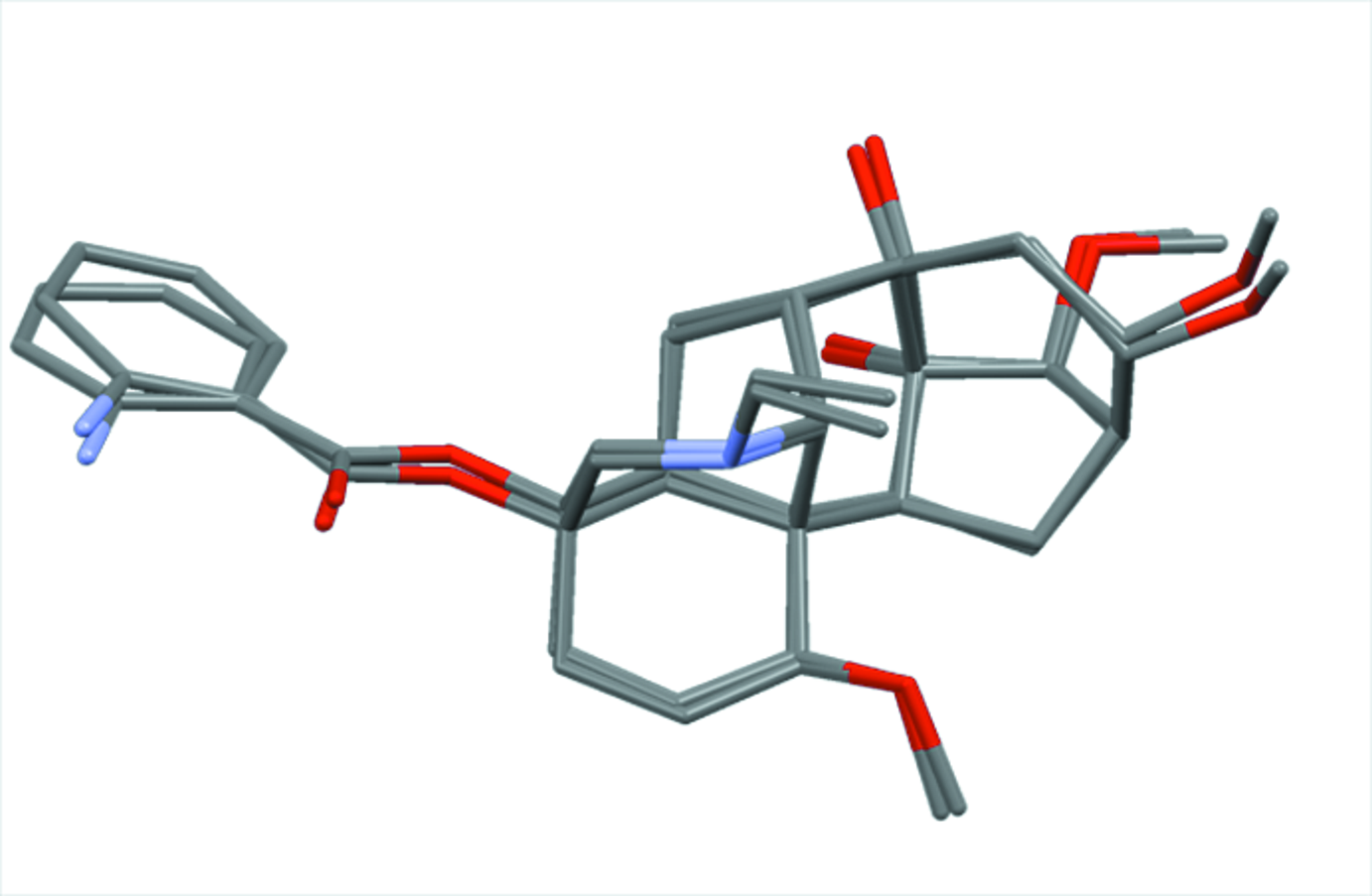

Supplement: Supplementary file 5 [file e-71-0o576-fig2.tif]
